# Editorial: Impact of Diet on Learning, Memory and Cognition

**DOI:** 10.3389/fnbeh.2017.00096

**Published:** 2017-05-19

**Authors:** Amy C. Reichelt, R. Fred Westbrook, Margaret J. Morris

**Affiliations:** ^1^School of Health and Biomedical Sciences, RMIT UniversityMelbourne, VIC, Australia; ^2^School of Psychology, University of New South WalesSydney, NSW, Australia; ^3^School of Medical Science, University of New South WalesSydney, NSW, Australia

**Keywords:** diet, obesity, cognition, memory, reward, adolescent, fMRI neuroimaging, behavior

The so-called western diet is rich in saturated fat and refined carbohydrates. Excessive consumption of this diet is associated not only with the development of obesity but also with reduced global cognitive function, cognitive decline, and dementia (Morris et al., 2015). This Research Topic explores the effects of diet and diet-induced obesity on learning, memory, and cognition in both experimental and epidemiological settings.

High fat and high sugar foods are highly rewarding and excessive consumption leads to enduring alterations in brain regions involved in learning, memory, and reward. These changes are proposed to drive overconsumption by promoting food seeking behaviors. Moreover, alterations in brain regions essential for learning, memory, and behavioral control induced by this diet appear to be especially profound in the immature brain (Boitard et al.; Gainey et al.; Reichelt).

The neuroplasticity mechanisms that underpin cognitive and behavioral alterations are reviewed by Morin et al., with particular reference to neuronal alterations in the hippocampus and prefrontal cortex (PFC), brain regions essential for encoding memories and controlling behavior, as well as in the amygdala and nucleus accumbens, regions involved in processing and seeking rewards. The abundance of palatable foods in modern environments contributes to their overconsumption, increases in body weight and progression to obesity. Kendig et al. examined whether food-seeking behaviors in rodents differ in an environment associated with junk foods vs. one that contained regular chow. The important result was that food seeking behavior in the environment associated with junk food became relatively inflexible and habit based, whereas food seeking in the chow associated environment was flexible and goal-directed. These and other findings (e.g., Furlong et al., 2014) may provide new insights into environmental determinants of over-consumption. Stressful experiences are also involved in triggering overconsumption in binge-eating disorder (BED). Lyu and Jackson utilized functional imaging (fMRI) following exposure to an acute stressor (cold pressor test) and observed reduced inhibitory hippocampal responsiveness to food cues in BED-symptomatic women.

Consumption of a western style diet in rats altered levels of the neurotransmitter dopamine and associated metabolites in the striatum and hippocampus, suggesting a mechanistic basis by which such diets may alter food related learning and memory processes (Nguyen et al.). Recent evidence has begun to link the gut microbiome with dietary- and metabolic-associated hippocampal impairment. High fat and/or high sugar diets alter gut bacteria (microbiotal) colonies and in turn increase intestinal permeability and reduce blood brain barrier integrity (Noble et al.). This creates a vulnerability to the influx of toxins from the circulation to the brain, potentially underpinning diet-induced cognitive dysfunction.

This Research Topic contains epidemiological and experimental evidence of sensitive or critical time points at which diet can alter brain and cognitive development with enduring consequences. For example, childhood obesity is increasingly prevalent and is associated with diminished cognition. Reichelt reviews preclinical evidence that exposure to high fat and high sugar diets during adolescence produces more profound cognitive deficits than such exposure during adulthood. Such diets may be especially injurious to cognition when consumed across adolescence because this is a period of heightened neuroplasticity due to age-specific maturational processes, including pruning of dopamine receptors in the prefrontal cortex.

Studies exploring the reversibility of diet-induced cognitive deficits in young animals through both dietary and pharmacological methods are presented in this Research Topic. Gainey et al. demonstrated that administration of glyburide, a second-generation drug for the treatment of type-2-diabetes that stimulates insulin release, attenuated high fat diet evoked deficits in anxiety and memory in young mice. Acute glyburide administration reversed high fat diet evoked memory deficits in a novel object recognition memory task and alleviated anxiety-like behaviors in mice fed a high fat diet across adolescence. Drugs that stimulate insulin secretion may thus have potential for the treatment of obesity-associated cognitive dysfunction. Furthermore, Boitard et al. showed that switching rodents to a standard chow diet for 12 weeks following adolescent consumption of a high fat diet for 12 weeks was sufficient to restore aspects of diet induced changes in cognitive and emotional processing. Rats returned to the chow diet exhibited greater hippocampal neurogenesis measured by doublecortin immunoreactivity, and reduced HPA axis reactivity measured by blood corticosterone, and amygdala activity by c-Fos, as well as better conditioned odor avoidance memory.

Poor diet is a potential risk factor for the development of cognitive impairment; conversely, dietary nutrients are protective against such impairments. Lu et al. reported a cross-sectional study which examined the impact of dietary nutrients on the development of mild cognitive impairment (MCI). Dietary intake of nutrients was compared between MCI patients and cognitively normal subjects. Carotenoids, vitamin C, and vitamin B6 were identified as the dietary nutrients with the highest protective capacity against MCI, potentially due to their antioxidant properties. Moreover, adequate dietary intake of monounsaturated fatty acids and cholesterol were significantly associated with decreased risk of MCI. Wang et al. examined the association between widespread scarcity of food at various childhood developmental stages (fetal exposure—late childhood exposure) on subsequent cognitive performance in an adult Chinese cohort (age range 51–65). These investigators found that famine during the fetal period was associated with subsequent global cognitive decline and increased risk of MCI, and that famine during mid- and late-childhood was associated with deficits in executive function in adulthood. Critically, this study highlights the importance of nutrient availability during early life on adult cognitive function.

What is apparent from the studies presented in this Research Topic is the pervasive influence of diet and food-associated environments on cognition, motivation, and behavioral control. The papers collected in this Research Topic offer new and valuable insights into the psychological processes and neural mechanisms underpinning this pervasive influence of diet and food-associated environments, be it through excessive consumption of fat and sugar, or malnutrition across the lifespan. The findings reported will form the basis for novel theoretical ideas and applications to an increasingly severe public health issue.

## Author contributions

AR, RW, and MM wrote the editorial and served as editors for the Research Topic.

## Funding

AR is the recipient of an Australian Research Council Discovery Early Career Research Award (DE140101071). MM and RW receive funding from the National Health and Medical Research Council, Australia (APP1126929).

### Conflict of interest statement

The authors declare that the research was conducted in the absence of any commercial or financial relationships that could be construed as a potential conflict of interest.
